# Household Pesticides and the Risk of Wilms Tumor

**DOI:** 10.1289/ehp.9298

**Published:** 2006-09-07

**Authors:** Maureen A. Cooney, Julie L. Daniels, Julie A. Ross, Norman E. Breslow, Brad H. Pollock, Andrew F. Olshan

**Affiliations:** 1 Department of Epidemiology, University of North Carolina, Chapel Hill, North Carolina, USA; 2 Department of Pediatrics, University of Minnesota, Minneapolis, Minnesota, USA; 3 Department of Biostatistics, University of Washington, Seattle, Washington, USA; 4 Department of Epidemiology and Biostatistics, University of Texas Health Science Center at San Antonio, San Antonio, Texas, USA

**Keywords:** childhood cancer, environment, pediatric pesticides, Wilms tumor

## Abstract

**Background:**

Previous epidemiologic studies have suggested that exposure to pesticides *in utero* and during early childhood may increase the risk for development of childhood cancer, including Wilms tumor, a childhood kidney tumor.

**Objectives:**

In this analysis we evaluated the role of residential pesticide exposure in relation to the risk of Wilms tumor in children using data from a North American case–control study.

**Methods:**

The National Wilms Tumor Study Group (NWTSG) collected information on exposure to residential pesticides from the month before pregnancy through the diagnosis reference date using detailed phone interviews from 523 case mothers and 517 controls frequency matched on child’s age and geographic region and identified by list-assisted random digit dialing. Pesticides were grouped according to type of pesticide and where they were used.

**Results:**

A slightly increased risk of Wilms tumor was found among children of mothers who reported insecticide use [odds ratio (OR) = 1.4, 95% confidence interval (CI), 1.0–1.8; adjusted for education, income, and the matching variables]. Results from all other categories of pesticides were generally close to the null.

**Conclusions:**

This study is the largest case–control study of Wilms tumor to date. We were unable to confirm earlier reports of an increased risk for Wilms tumor among those exposed to residential pesticides during pregnancy through early childhood.

Wilms tumor (nephroblastoma) is an embryonal malignancy of the kidney. It is one of the most common abdominal malignancies of childhood. In the United States, approximately 500 children < 20 years of age are diagnosed with Wilms tumor each year. The incidence of Wilms has remained relatively unchanged over the 21 years from 1975 to 1995 (Ries et al. 1999).

Wilms tumor is usually diagnosed before the child reaches 5 years of age (Ries et al. 1999). The younger age at diagnosis of Wilms tumor highlights the need to explore early life exposures, particularly those occurring around conception through early childhood. Pesticides are of concern because of the great potential for children to be exposed to them in the home. Children spend a lot of time with pets, on the floor, and in the yard—all places where pesticide use can be high (Davis et al. 1992; Fenske et al. 1990; Garry 2004; Lewis et al. 1994; Morgan et al. 2005). Studies have investigated associations between parental pesticide exposure in agricultural, occupational, and residential settings and the risk of Wilms tumor, with mixed results (Fear et al. 1998; Kristensen et al. 1996; Olshan et al. 1993; Sharpe et al. 1995; Tsai et al. 2006; Wilkins and Sinks 1984). One study reported an association between household extermination and Wilms tumor (Olshan et al. 1993), whereas another study reported no association between residential pesticides and Wilms tumor (Wilkins and Sinks 1984). The most recent study (Tsai et al. 2006) examined pesticide exposure through maternal questionnaire, reporting an odds ratio of 1.3 [90% confidence interval (CI) 0.8–2.0] for maternal pesticide use during pregnancy. To explore further the association between residential pesticide use and the development of Wilms tumor, we conducted a large case–control study that included detailed information on residential pesticide exposure.

## Materials and Methods

Cases were patients newly diagnosed with Wilms tumor < 16 years of age and treated at one of 128 participating hospitals in the United States and Canada from 1999 to 2002. Eligible cases included all newly diagnosed patients registered with the National Wilms Tumor Study (NWTS), which is a North American collaborative clinical trial study of the treatment and biology of Wilms tumor. The NWTS Group included members of either of the two pediatric collaborative clinical trials groups, the Children’s Cancer Group and the Pediatric Oncology Group (now merged into the Children’s Oncology Group). The coverage for ascertainment by the NWTS has been shown to be very high, approximating 95% (Ross et al. 1996). The treating institution obtained institutional review board (IRB) approval for the study, and the primary physician provided permission to contact the family. Cases were excluded if they had clear cell sarcoma or rhabdoid tumor of the kidney. In addition, cases were eligible if the biological mother spoke either English or Spanish and they had a telephone in the home. Five hundred twelve identified potential cases were not recruited because of the lack of institution IRB approval or because the primary physician did not allow the study to contact them. Using data from the NWTS-5 clinical database (Dome et al. 2006), we evaluated sex, race, survival, stage, and geographic region and found no significant differences between the interviewed cases and noninterviewed eligible cases. Of the 653 eligible cases, 523 case mothers (80%) were successfully interviewed. Reasons for nonparticipation included lost-follow-up (*n* = 58), refusal (*n* = 47), and other reasons (*n* = 25). Controls were identified through list-assisted random-digit dialing (RDD) and were frequency matched to cases according to age at diagnosis using 3 age strata (0–1, 2–3, and ≥ 4 years) and geographic region of residence using five location strata (four U.S. Census regions plus Canada). The response proportion was 51% for the RDD screening phase. Of the 682 eligible control mothers identified, 517 (76%) successfully completed interviews. Because of the poor response among control fathers (< 50%), we focused only on maternal interview data.

Structured computer-assisted telephone interviews were conducted with the mothers of cases and controls after initial contact was made and signed consent forms were received. We used the case diagnosis date as the reference date for cases. and the initial RDD screening date as the reference date for controls. During the interview, information was collected on potential risk factors for Wilms tumor including demographic factors, pregnancy history, birth characteristics, childhood exposures, parental occupational history, family medical history, and use of tobacco, alcohol, and medication.

The interview included a section on household pesticide use. Mothers were asked about the use of chemical products to control different pests from the month before pregnancy with the index child through the reference date. Residential pesticides were defined as chemical products used to control insects, fungus, rodents, and weeds, in the house or yard, insects on pets, insects on the parent or child’s body, and professional extermination of the home or lawn. If the mother answered yes, she was asked how many times she used the product in specific time periods reflecting the month before pregnancy through the pregnancy and after the child was born up until the reference date.

In the analysis, pesticides were categorized by the location where the chemicals were used (i.e., house, lawn, child, pet, or mother’s body), by type (i.e., insecticide, herbicide, fungicide, rodenticide), and by combination of location and type (e.g., household insecticide). We then evaluated pesticide use in the exposure time windows (month before pregnancy through the pregnancy, birth through the reference date).

We estimated odds ratios (ORs) and 95% CIs for the association between pesticide use and Wilms tumor using unconditional logistic regression models. We evaluated potential confounders, including household income, maternal education, breast-feeding, and maternal age, in combination with the frequency matching variables (child’s age at reference date and geographic region of residence). We used four models to determine whether the hypothesized confounders should be included in the final model. Model 1 included maternal age and breast-feeding and the matching variables as potential confounders; model 2 included income, education, maternal age, breast-feeding, and the matching variables; model 3 included education, income, and the matching variables; model 4 included only the matching variables. None of the combinations of these variables materially changed the crude pesticide effect estimates. The final model included the matching variables and two *a priori* confounders, education and income, selected as potential surrogates of selection processes related to RDD and nonresponse. We also evaluated effect measure modification based on the significance (*p* < 0.10) of the likelihood ratio test of the interaction term in the logistic regression model. Factors examined as potential effect measure modifiers included reference age at diagnosis, mother’s education, and breast-feeding.

## Results

This analysis was based on information obtained in telephone interviews from 523 case mothers and 517 control mothers. We found that cases and controls and their mothers were similar with regard to basic demographic characteristics (Table 1). There was some indication that children of mothers in a household with an income < $10,000 were at slightly higher risk of Wilms tumor, but the OR was not very precise. A difference in the distribution of sex was found between the two groups with a higher percentage of female cases (57.2%) compared with controls (47.2%). African Americans and Hispanics had a slightly higher risk for Wilms tumor, although these estimates were imprecise due to small numbers.

Approximately 61% of the case mothers and 57% of control mothers reported using pesticides (Table 2). Overall, pesticide use was associated with a slight increase in risk of Wilms tumor, after adjustment for the matching variables, income and education (OR = 1.3; 95% CI, 1.0–1.7). This result may represent insecticide use, because 88% of those noting pesticide use reported using insecticides. The association between any insecticide use and Wilms tumor was similar (OR = 1.4; 95% CI, 1.0–1.8). Insecticides were used more commonly than other types of pesticides; yet 33% of women reported using more than one pesticide type. Elevated ORs were not found for herbicides or fungicides.

We evaluated pesticides by type and place of use. Most were not associated with Wilms tumor; however, the use of any type of pesticide in the house was associated with a small increased risk of Wilms tumor (OR = 1.4; 95% CI, 1.0–1.8). This association was the same for insecticide use in the house. ORs associated with insecticides used in the yard or on the skin were slightly attenuated. Results for the frequency of pesticide use were generally imprecise, null, and did not reveal dose–response patterns. For insecticide use in the home, the magnitude of the OR was greater, but much less precise, for least frequent use (once per 7–12 months, OR = 2.1; 95% CI, 1.1–3.8) and most frequent use (once per month, OR = 2.2; 95% CI, 1.1–4.4) than for other frequency of use categories; this did not support a dose–response pattern.

The results were essentially the same regardless of whether the exposure occurred during the pregnancy or during the childhood period (data not shown). In addition, the associations appeared similar among children who were breast-fed and those who were not. Most estimates were close to the null and imprecise, because of the reduced sample size in each stratum. Although ORs varied slightly by strata, none of the factors examined as effect measure modifiers were statistically significant (*p* < 0.10) on the basis of the likelihood ratio test. For example, the effect of any pesticide use by child’s age at diagnosis was 0–1 year of age (adjusted OR = 1.3; 95% CI, 0.8–2.2); 2–3 years of age (OR = 1.5; 95% CI, 0.9–2.4); and ≥ 4 years of age (OR = 1.1; 95% CI, 0.7–1.7).

Child’s sex was also examined as a potential effect modifer and a confounder. Sex was not an important confounder when included in our models, and when results were stratified by sex, no important differences were found. For example, the OR for insecticide use was 1.3 (95% CI, 0.9–1.9) among males and 1.4 (95% CI, 0.9–1.9) among females.

## Discussion

Exposure to residential pesticides did not produce a strong pattern of increased risk of developing Wilms tumor in this study. We classified the pesticides by intended use as a proxy of their chemical makeup. This division was important to distinguish whether any particular pesticide group conveyed more risk than another. Except for slightly higher risk associated with insecticides, most associations between pesticides and Wilms tumor were weak. There was also no apparent elevation in risk associated with exposure during different time periods before compared with after the birth of the child, nor modification of the effects of pesticides by child’s age or sex.

The first NWTS study found an association with household insect extermination (OR = 2.16; 95% CI, 1.24–3.75) (Olshan et al. 1993). Our study found no association between extermination and Wilms tumor. The more detailed data collection through structured telephone interview for the current study may have better distinguished pesticide usage patterns compared with the earlier study’s broad self-administered mailed questionnaire with less detailed questions.

This is the largest case–control study of risk factors for Wilms tumor conducted to date and has detailed exposure information using computerized telephone interviews. Women were queried about the types of pesticides used and the timing and location of their use. Mothers were asked to remember their use of pesticides up to several years before the interview. Recall could be problematic because use of chemicals in and around the house was common and memory of details over time could be inaccurate. One recent study found a high correlation for residential pesticides between self-reported pesticide exposure and household dust samples (Hartge et al. 2005). However, another study suggested that under-reporting is a common problem with self-reported pesticide exposure from surveys (Nieuwenhuijsen et al. 2005). According to a U.S. Environmental Protection Agency report, in 1999–2001 in the United States the three most commonly used pesticides outside of the agricultural market were glyphosate, atrazome, and metam sodium; the most common insecticide used was malathion (Kiely et al. 2004). However, because mothers were not asked to recall the specific type of pesticide they used, we had limited ability to evaluate specific chemicals.

When pesticide use was reported, we did not ascertain whether the mother took any preventive steps such as wearing protective gear, ventilating the space, or removing the child from the room while using the pesticide—which would affect the level of exposure. A study of childhood brain tumors reported elevated risk when these exposure mediators were considered (Pogoda and Preston-Martin 1997).

Another limitation of our study was our inability to assess father’s report of residential pesticide use because of the sparse nature of the data. Different risk pathways may be operating depending on whether the mother or the father is exposed. Mothers and fathers may differentially recall the use of pesticides in or around the home (Daniels et al. 2001). It is unclear which parent better reports pesticide use, but some reporting error may be based on who applied the pesticides. The exposure level may differ for the applicator compared with other members of the household. The resulting exposure misclassification may be differential or nondifferential, and predictions about the direction of the bias are difficult to infer.

The control participation proportion (76%) was good, but the RDD screening proportion was low. Only 50% of those households contacted agreed to complete the screening questionnaire. It is difficult to speculate about the direction or magnitude to which this bias may have affected our results because we do not know the characteristics of those who did not respond to the initial request. However, we do know that those who participated were not different in demographic characteristics from those families who completed the screen but then decided not to participate.

Although the association between residential insecticide use and Wilms tumor may warrant further investigation, overall these findings do not support other positive findings from studies of residential or occupational pesticide exposures (Fear et al. 1998; Kristensen et al. 1996; Olshan et al. 1993; Sharpe et al. 1995). It has been suggested that preconceptual exposure to pesticides may play a role in the development of Wilms tumor by causing damage to parental germ cells (Tsai et al. 2005). We were unable to assess the potential for preconceptual exposure of fathers in this study, and most fathers were not likely to have exposure to pesticides at a level comparable to those of occupationally exposed workers. The ability of future studies to better investigate the relation between pesticides and Wilms tumor directly relies on whether advanced technology is available to improve exposure assessment, possibly using dust or biologic samples, to reflect more accurately the exposure during relevant time windows before and during pregnancy.

## Figures and Tables

**Table 1:** Maternal and child demographic characteristics [no. (%)] and associated ORs (95% CIs) for children diagnosed with Wilms tumor and age/region-matched controls, United States and Canada, 1999–2002.

Maternal and child characteristics	Cases (*n* = 523)^a^ No. (%)	Controls (*n* = 517)^a^ No. (%)	OR (95% CI)^b^
Child’s age at reference date (years)			
0–1	146 (27.9)	138 (26.7)	1.2 (0.9–1.6)
2–3	165 (31.6)	145 (28.1)	1.3 (0.9–1.7)
≥ 4	212 (40.5)	234 (45.3)	1.0
Geographic region of residence			
Midwest	159 (30.4)	154 (29.8)	1.0
Northeast	69 (13.2)	60 (11.6)	1.1 (0.7–1.7)
South	180 (34.4)	183 (35.4)	1.0 (0.7–1.3)
West	58 (11.1)	64 (12.4)	0.9 (0.6–1.3)
Canada	57 (10.9)	56 (10.8)	1.0 (0.7–1.6)
Mother’s education			
0–11 years	45 (8.6)	41 (7.9)	1.0
High school	137 (26.2)	118 (22.8)	1.2 (0.7–2.0)
> High school	341 (65.2)	357 (69.1)	0.8 (0.5–1.3)
Household income at birth (US$)			
< 10,000	47 (9.0)	38 (7.4)	1.3 (0.7–2.4)
10,000–20,000	81 (15.5)	96 (18.6)	0.9 (0.6–1.4)
21,000–30,000	65 (12.4)	67 (13.0)	0.9 (0.5–1.4)
31,000–40,000	66 (12.6)	66 (12.8)	1.0
41,000–50,000	64 (12.2)	63 (12.2)	1.0 (0.6–1.6)
≥ 51,000	158 (30.2)	146 (18.3)	1.0 (0.7–1.6)
Mother’s race			
White	390 (74.6)	404 (78.1)	1.0
Black	73 (14.0)	58 (11.2)	1.4 (0.9–2.0)
Hispanic	42 (8.0)	33 (6.4)	1.4 (0.8–2.3)
Other	18 (3.4)	22 (4.3)	0.9 (0.5–1.6)
Mother’s age at child’s birth (years)			
< 20	40 (7.7)	39 (7.5)	0.8 (0.5–1.4)
20–24	114 (21.8)	103 (19.9)	1.0
25–30	182 (34.8)	164 (31.7)	1.0 (0.7–1.4)
≥ 31	187 (35.8)	211 (40.8)	0.8 (0.6–1.1)
Sex			
Male	224 (42.8)	273 (52.8)	1.0
Female	299 (57.2)	244 (47.2)	1.5 (1.2–1.9)

a Among cases, there were 42 missing values for household income. Among controls, there was one missing value for mother’s education and 41 missing values for household income.

b ORs adjusted by child’s age at reference date and geographic region of residence (matching factors).

**Table 2:** ORs (95% CIs)^a^ for risk of Wilms tumor associated with exposure^b^ to pesticides, NWTS 1999–2002.

	Cases [no. (%)]		Controls [no. (%)]		
	Exposed	Unexposed	Exposed	Unexposed	OR (95% CI)
Pesticide	320 (61.2)	203 (38.8)	292 (56.5)	225 (43.5)	1.3 (1.0–1.7)
In home	176 (34.5)	334 (65.5)	148 (29.5)	353 (70.5)	1.3 (1.0–1.7)
In yard	158 (31.0)	352 (69.0)	157 (31.3)	344 (68.7)	1.0 (0.8–1.4)
On body	148 (29.0)	362 (71.0)	147 (29.0)	354 (71.0)	1.0 (0.8–1.4)
Insecticide	285 (54.5)	238 (45.5)	251 (48.6)	266 (51.5)	1.4 (1.0–1.8)
In home	154 (30.2)	356 (69.8)	123 (24.6)	378 (75.5)	1.4 (1.0–1.8)
In yard	103 (20.2)	406 (79.8)	94 (18.9)	404 (81.1)	1.2 (0.8–1.6)
On mother	80 (17.5)	420 (82.5)	89 (16.0)	421 (84.0)	1.2 (0.8–1.7)
On child	91 (18.0)	415 (82.0)	82 (16.7)	409 (83.3)	1.2 (0.8–1.7)
Herbicides	112 (21.4)	411 (78.6)	112 (21.7)	405 (78.3)	1.0 (0.7–1.4)
Fungicides	32 (6.1)	491 (93.9)	31 (6.0)	486 (94.0)	1.0 (0.6–1.7)
In home	27 (5.3)	483 (94.7)	22 (4.4)	479 (95.6)	1.2 (0.7–2.1)
In yard	6 (1.2)	500 (98.8)	10 (2.0)	490 (98.0)	0.7 (0.2–1.9)
Rodenticides	20 (3.9)	490 (96.1)	19 (3.8)	482 (96.2)	1.0 (0.5–2.0)
Exterminator	145 (28.6)	362 (71.4)	149 (29.9)	349 (70.1)	1.0 (0.7–1.3)

For the broadest categorization of the pesticides into herbicides, fungicides, insecticides, there were no missing data. Some data were missing for 13 cases and 16 controls when pesticides were classified based on both type and location of use.

a Adjusted for age at reference date, geographic region, education, and income.

b Exposure during any time from pregnancy through childhood.
